# Enhanced Electrical Performance and Stability of Solution-Processed Thin-Film Transistors with In_2_O_3_/In_2_O_3_:Gd Heterojunction Channel Layer

**DOI:** 10.3390/nano12162783

**Published:** 2022-08-14

**Authors:** Shasha Li, Xinan Zhang, Penglin Zhang, Guoxiang Song, Li Yuan

**Affiliations:** 1 College of Engineering Physics, Shenzhen Technology University, Shenzhen 518118, China; 2 School of Physics and Electronics, Henan University, Kaifeng 475004, China

**Keywords:** solution process, TFTs, doping, heterojunction channel layer, mobility, stability

## Abstract

The use of the semiconductor heterojunction channel layer has been explored as a method for improving the performance of metal oxide thin-film transistors (TFTs). The excellent electrical performance and stability of heterojunction TFTs is easy for vacuum-based techniques, but difficult for the solution process. Here, we fabricated In_2_O_3_/In_2_O_3_:Gd (In_2_O_3_/InGdO) heterojunction TFTs using a solution process and compared the electrical properties with single-layer In_2_O_3_ TFTs and In_2_O_3_:Gd (InGdO) TFTs. The In_2_O_3_/InGdO TFT consisted of a highly conductive In_2_O_3_ film as the primary transmission layer and a subconductive InGdO film as the buffer layer, and exhibited excellent electrical performance. Furthermore, by altering the Gd dopant concentration, we obtained an optimal In_2_O_3_/InGdO TFT with a higher saturation mobility (µ) of 4.34 cm^2^V^−1^s^−1^, a near-zero threshold voltage (V_th_), a small off-state current (I_off_) of $1.24\times 10^{-9}$ A, a large on/off current ratio (I_on_/I_off_) of $3.18\times 10^{5}$, a small subthreshold swing (SS), and an appropriate positive bias stability (PBS). Finally, an aging test was performed after three months, indicating that In_2_O_3_/InGdO TFTs enable long-term air stability while retaining a high-mobility optimal switching property. This study suggests that the role of a high-performance In_2_O_3_/InGdO heterojunction channel layer fabricated by the solution process in the TFT is underlined, which further explores a broad pathway for the development of high-performance, low-cost, and large-area oxide electronics.

## 1. Introduction

Metal oxide thin-film transistors (MO TFTs) have attracted considerable attention because of their high carrier mobilities, excellent optical transparency, good environmental stability, and low temperature processing in active-matrix liquid crystal displays (AMLCD), active-matrix organic light-emitting diode (AMOLED) displays, and other optoelectronic devices [1,2,3]. At present, various metal oxides have been extensively investigated as channel layers for TFTs and exhibit a superior performance, such as In_2_O_3_, ZnO, InZnO, InGaZnO, etc. [4,5,6,7,8]. Among them, In_2_O_3_ is widely considered to be an ideal candidate to fabricate high-electrical-performance TFTs, owing to its wide band gap, low temperature processability [9], and high field effect carrier mobility due to the efficient percolation pathway of the In 5s orbital. However, the low on/off current ratio (I_on_/I_off_), large subthreshold swing (SS), large negative threshold voltage (V_th_), and poor bias reliability in the intrinsic In_2_O_3_ TFTs limit their industrial applications in the next generation of display technologies [10,11]. To overcome this issue, the proper dopant as a carrier suppressor is indispensable [12]. Choosing an appropriate dopant depends generally on electronegativity; the large difference in electronegativity between the dopant and oxygen helps form strong metal–oxygen bonds [13], which is the critical factor for enhancing the stability and performance of the MO TFTs [12]. Nowadays, the effects of carrier suppressors such as Ga, Zr, Hf, Gd, Y and Al on thin films and devices were broadly investigated [14,15,16,17]. Additionally, the results reveal that the carrier suppressors can help reduce the instability because they can effectively repress the generation of oxygen vacancy by binding strongly to oxygen [8,12,17,18,19] Recently, Gd has been selected as a superb dopant candidate in view of its lower electronegativity of 1.20 compared with indium [13]. This is consistent with the earlier research that incorporated Gd in In_2_O_3_ to significantly improve the electrical properties of In-based TFTs [13]. Nevertheless, the incorporation of carrier suppressors in the semiconducting film leads to the degradation of mobility because oxygen vacancies also act as carrier electron donors through their ionization; simultaneously, the threshold voltage shifts to higher values [20]. Recently, a heterojunction channel structure was successfully implemented by combining two different compositions of semi-conducted thin-film channel layers, such as IGZO/IGZO: Ti [21], VZTO/ZTO [20], InZnO/AlSnZnInO [22], In_2_O_3_/ZnO [5], AZO/ZO [16], ZnO/IGO [23], In_2_O_3_/IGZO [24], ZnO/In_2_O_3_ [25], and MgZnO/ZnO [26]. These studies indicate that the heterojunction channel layer comprising a highly conductive layer with high mobility and the semiconducting layer with low I_off_ and high stability have been resolved to upgrade the electrical performance and stability [4,26,27,28]. In general, the predominant techniques for depositing oxide films are vacuum-based techniques, such as RF magnetron sputtering, atomic layer deposition, pulse laser deposition, etc. [7,29,30]. These pose some limitations in mass production and the realization of low-cost electronic devices. Fortunately, the solution process has been extensively employed for simplicity, the low-cost fabrication of large areas, easy controllability of chemical stoichiometry, and mass productivity [3,31,32]. Excellent electrical performance and stability of TFTs with the heterojunction channel layer would be easy for the vacuum sputtering deposition process, but difficult for the solution process. Some attempts in this direction have not shown significant improvement in contrast to single-layer channel TFTs.

In this paper, we investigated the impact of Gd doping on the electrical performance of the In_2_O_3_ TFTs by the solution process. Then, the In_2_O_3_/InGdO TFTs were constructed and contrasted to single-layer In_2_O_3_ or InGdO TFTs to validate the vantages of the bilayer structure. Finally, we optimized the electrical performance of the In_2_O_3_/InGdO TFTs by varying the Gd doping concentration and reported a comprehensive high performance TFT with a higher mobility of 4.34 cm^2^V^−1^s^−1^, a near-zero V_th_, a small I_off_ of $1.24\times 10^{-9}\;$ A, a large I_on_/I_off_ of $3.18\times 10^{5}$, a small SS and an appropriate PBS. Finally, an aging test was carried out after three months, indicating the excellent environmental stability of the devices.

## 2. Experimental Section

The In_2_O_3_ precursor solution was prepared by dissolving 99.99% purity Indium nitrate hydrate (In(NO3)3.xH2O) in 2-methoxyethanol to yield a 0.05 M solution. InGdO precursor solution with different Gd doping concentrations was separately prepared by dissolving 99.99% purity In(NO3)3.xH2O in 2-methoxyethanol with moderate gadolinium nitrate hexahydrate (Gd(NO3)3.6H_2_O) to yield a 0.05 M solution. All reagents were purchased from Aladdin (Shanghai, China) and used without further purification. Precursor solutions were stirred overnight at room temperature, and then filtered through a 0.2 μm PTFE membrane filter before spin coating.

**Figure 1:**
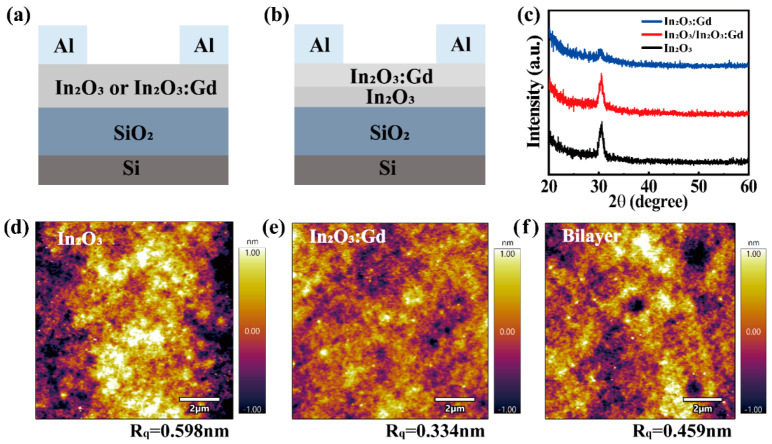
Schematic cross-sectional view of (**a**) the In_2_O_3_ or InGdO and (**b**) the In_2_O_3_/InGdO heterojunction TFTs. (**c**) XRD patterns of the In_2_O_3_, InGdO, and In_2_O_3_/InGdO thin films annealed at 300 °C. AFM surface morphology of (**d**) In_2_O_3_ (**e**) InGdO (**f**) In_2_O_3_/InGdO thin films annealed at 300 °C.

The TFTs were fabricated on n-type Si substrates with thermally grown SiO_2_ (300 nm) as the gate insulator. Before the active layer deposition, the substrate was ultrasonically cleaned in acetone, ethanol and deionized water, sequentially, for 10 min each, and dried it using high purity N_2_. To improve the chemical compatibility between the interface of the SiO_2_ and the semiconductor layer, the surface of the cleaned substrate was hydrophilized for 10 min with O_2_ plasma. For the In_2_O_3_/InGdO TFTs, the In_2_O_3_ precursor solution was filtered, spin-coated onto the SiO_2_ at 3000 rpm for 30 s, and then annealed on a hotplate at 300 °C for 20 min. The thicknesses of the In_2_O_3_ layer were controlled by repeating the procedure. Subsequently, the InGdO precursor solution was immediately spin-coated onto the In_2_O_3_ film to avoid contamination and annealed under the same conditions. The In_2_O_3_ and InGdO films have a similar thickness of about 10 nm measured by surface profilometer. Finally, the 80 nm Al source and drain electrodes were thermally evaporated onto the semiconductor layer through a shadow mask that has a channel width (W) and length (L) of 600 and 100 µm, respectively. Similarly, the single-layer In_2_O_3_ or InGdO TFTs were fabricated for comparison. The schematics cross-sectional view of the single-layer In_2_O_3_ or InGdO and the In_2_O_3_/InGdO heterojunction TFTs are shown in Figure 1a,b. The crystallinity of the films was investigated by X-ray diffraction (XRD, Karlsruhe, Germany) with Cu Kα radiation. The surface morphologies and roughness of the films were studied by atomic force microscopy (AFM, Santa Barbara, CA, USA). The electrical characterization of TFTs was performed by using the semiconductor parameter analyzer (Keithley 2612B, Cleveland, OH, USA).

## 3. Result and Discussion

Figure 1a,b show the schematic cross-sectional view of the TFTs. The crystallinities of the In_2_O_3_, InGdO and In_2_O_3_/InGdO films annealed at the identical temperature were measured by XRD, as shown in Figure 1c. A nanocrystalline structure with a relatively sharp dominant diffraction peak corresponding to the (222) plane was observed in the In_2_O_3_ film. On the other hand, the peak intensity of the InGdO film was significantly diminished compared with that of the In_2_O_3_ film. It implies that Gd ions may lead to the degradation of the In_2_O_3_ crystallinity to a certain extent, which is consistent with the results of previous studies regarding the disappearance of the peak of InGdO films via Gd doping [13]. However, the diffraction peaks corresponding in the In_2_O_3_ and In_2_O_3_/InGdO films do not change significantly, which demonstrates that the thinness of the InGdO film cannot affect the underlying In_2_O_3_ film. The surface morphology of In_2_O_3_, InGdO, and In_2_O_3_/InGdO films at 300 °C were checked by AFM and shown in Figure 1d–f, respectively. Based on the AFM results, the root-mean-square (RMS) roughness of In_2_O_3_ films over a scan area of 10 μm × 10 μm was 0.598 nm. However, the InGdO film was featureless and smooth, and its RMS roughness was 0.334 nm less than that of In_2_O_3_ film. Presumably, the Gd ions in the InGdO film mitigated the degree of random crystallization, leading to the reduced RMS roughness value, which is consistent with the previous report. Additionally, the result was also confirmed from the XRD analysis, as mentioned above. Interestingly, the surface roughness of the In_2_O_3_/InGdO film was an equalization between the In_2_O_3_ and InGdO films, and its RMS roughness value was 0.495 nm, suggesting that the InGdO capping layer can alleviate In_2_O_3_ film roughening during the deposition annealing process at 300 °C.

**Figure 2:**
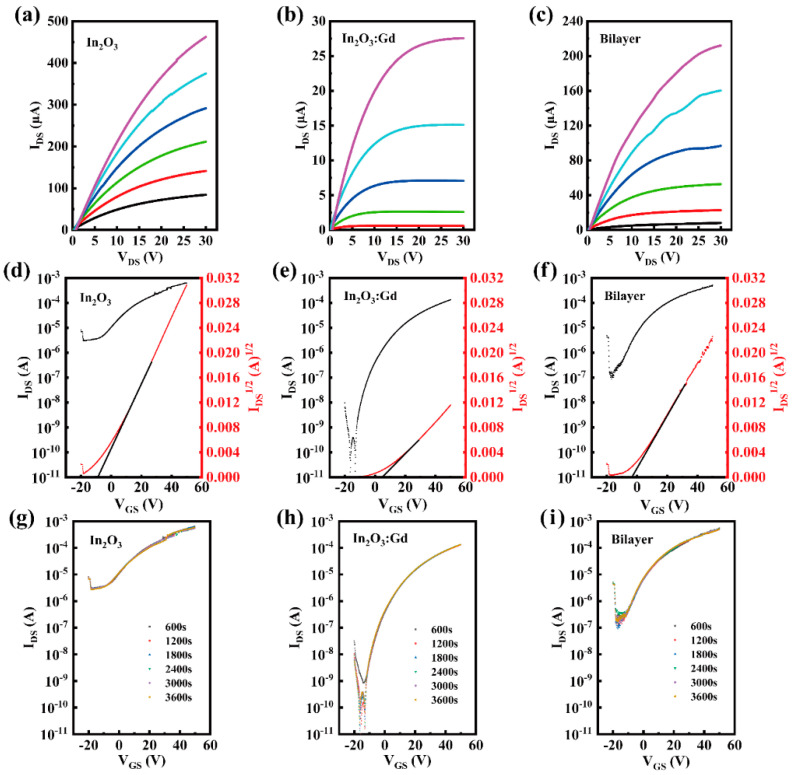
The representative output curves (**a**–**c**) and transfer curves (**d**–**f**) and transfer curves under the PBS (**g**–**i**) of the In_2_O_3_ or InGdO single-layer and In_2_O_3_/InGdO heterojunction TFTs at V_DS_ = 10 V.

The representative output and transfer curves of TFTs with In_2_O_3_ or InGdO single-layer and In_2_O_3_/InGdO heterojunction channel layer are shown in Figure 2a–f. The output characteristics of the TFTs were measured by changing the drain voltage (V_DS_) from 0 to 30 V at different constant gate voltages (V_GS_) varied from 0 to 30 V in a step of 6 V. The transfer characteristics of TFTs were measured at a V_DS_ of 10 V and a V_GS_ swing from −20~+50 V, whereas the source electrodes were fixed to 0 V. By a linear fit plot of the square root of the I_DS_ versus V_GS_, the mobility is obtained from the equation:

(1) $$I_{\mathrm{DS}}=\left(\frac{C_{i}\mu W}{2L}\right){\left(V_{\mathrm{GS}}-V_{\mathrm{th}}\right)}^{2}$$

where C_i_ is the capacitance per unit area of the SiO_2_ layer, and V_th_, V_GS_, W, and L denote the threshold voltage, the gate voltage, the channel width, and channel length, respectively. The V_th_ can be extracted by linear extrapolation of the I_D_^1/2^-V_GS_ at saturation regions. To further investigate the electrical properties of the TFTs, the subthreshold swing (SS) values are noticed and directly reflect the switching speed of the TFT devices. SS is directly related to the trap states at the interface of the dielectric layer to the metal oxide; it can be derived as follows:

(2) $$\mathrm{SS}={\left(\frac{\mathrm{dlog}\left(I_{\mathrm{DS}}\right)}{{\mathrm{dV}}_{\mathrm{GS}}}\right)}^{-1}\;\;\;\;$$

Based on Figure 2d–f, the In_2_O_3_ TFT exhibited a mobility of 9.34 cm^2^V^−1^s^−1^, V_th_ of −7.94 V, I_off_ of 2.89 × 10^−6^, and I_on_/I_off_ of 2.65 × 10^2^. As speculated, the InGdO TFT showed a significantly decreased I_off_ of 1.79 × 10^−11^A and a superior I_on_/I_off_ of 1.30 × 10^7^, which are desirable for practical applications. However, the on-state current (I_on_) was significantly reduced in the InGdO TFTs. Simultaneously, the InGdO TFT achieved a lower mobility of 1.37 cm^2^V^−1^s^−1^ and a positive offset V_th_, in comparison to In_2_O_3_ TFT. This is attributed to the formation of strong oxygen bonds between Gd ions and oxygen, which reduces mobility because the lack of oxygen vacancies prevents prefilling of trap states [1,13,33]. The V_th_ value for the InGdO TFT increased to approximately 5.89 V compared with that of the control In_2_O_3_ TFT. Additionally, the forward shift in V_th_ is caused by the need for a larger gate voltage to induce more carriers to prefill traps [1]. This follows the previous report that a more positive drift of V_th_ indicates a lower mobility in the channels [16]. Concurrently, the InGdO TFT exhibited an impressive switching speed; its SS was surprisingly improved to 1.75 V/dec compared with the In_2_O_3_ TFT with a very unsatisfactory SS. The tremendous enhancement in SS illustrates that the concentration of the traps at the SiO_2_/InGdO interface is greatly diminished for the InGdO TFT [34]. Interestingly, the In_2_O_3_/InGdO TFT presented a noteworthy mobility, V_th_, improved I_on_/I_off_, and acceptable I_off_ and SS, as shown in Figure 2f. When V_GS_  ≥  0 V, electrons near the InGdO layer are induced and aggregated to form a conductive channel. When the V_GS_ is more than the V_th_, the dominant current path formed by the electron aggregation between the insulating layer and the In_2_O_3_ layer creates a high-concentration carrier channel. The InGdO thin film plays a vital position as the buffer layer; it can drastically decrease the I_off_ of the In_2_O_3_/InGdO TFT. The In_2_O_3_ film provides enough current carriers; therefore, it enhances the I_on_/I_off_ of the device. It should be emphasized that the mobility value of the In_2_O_3_/InGdO TFT reaches up to 5.38 cm^2^V^−1^s^−1^, surpassing that of the InGdO TFT, which is accredited to the In_2_O_3_ layer with extremely high mobility. The I_on_/I_off_, SS, V_th_ and I_off_ values of In_2_O_3_/InGdO TFT were also improved to 9.01 × 10^3^, 7.68 V/dec, −3.77 V and 7.86 × 10^−8^ A, respectively, compared with those of the In_2_O_3_ TFT. The superior performance of the In_2_O_3_/InGdO TFTs strongly suggests that the In-based TFTs were effectively optimized without significantly sacrificing the carrier mobility by adopting the In_2_O_3_/InGdO heterojunction structure. It can be inferred that the InGdO capping layer on the In_2_O_3_ film further reduced the trap states through the controlled crystallization during the deposition annealing process, as mentioned in the XRD and AFM analyses, and is also partially responsible for the superior performance of the In_2_O_3_/InGdO heterojunction device.

The transfer curves for In_2_O_3_, InGdO, and In_2_O_3_/InGdO TFTs under positive bias stability (PBS) were investigated to analyze the electrical stability of the devices, respectively, as illustrated in Figure 2g–i. The threshold voltage shift was designated as ΔV_th_. The PBS of the TFTs were conducted with a consecutive 6-time sweep under V_GS_ swept in the range of −20 to +50 V and V_DS_ fixed at high positive bias of +10 V for a duration of 1 h at room temperature. From Figure 2g, the In_2_O_3_ TFT exhibits a high ΔV_th_ of 1.71 V, which is due to the numerous oxygen vacancies at the bulk region or the massive trap states at the SiO_2_/In_2_O_3_ interface [17,35]. In addition, it was reported that the interaction between the channel layer and oxygen in an ambient atmosphere plays a critical role in determining the V_th_ instability. When the PBS test was applied in the atmosphere, excess electrons accumulated in the channel layer and were captured by the surrounding oxygen molecules, which exhausts electron carriers and leads to the V_th_ positive shift [36]. The InGdO TFTs exhibited improved PBS stability of 0.85 V, which is attributed to the reduced oxygen vacancies at the interface and/or bulk region by the presence of Gd in In_2_O_3_ or the strong oxygen binders Gd prevent the desorption of oxygen from the atmosphere [17]. Additionally, the PBS property of In_2_O_3_/InGdO TFT was shown in Figure 2i. The device has an intermediate ΔV_th_ value of 1.26 V, which is essentially due to the total amount of oxygen vacancies obviously decreased from the In_2_O_3_ to the In_2_O_3_/InGdO; it is partially responsible for the superior PBT stability of the In_2_O_3_/InGdO TFTs. Moreover, it may also be one of the reasons that the strong oxygen binder Gd prevents the desorption of oxygen in the air.

**Figure 3:**
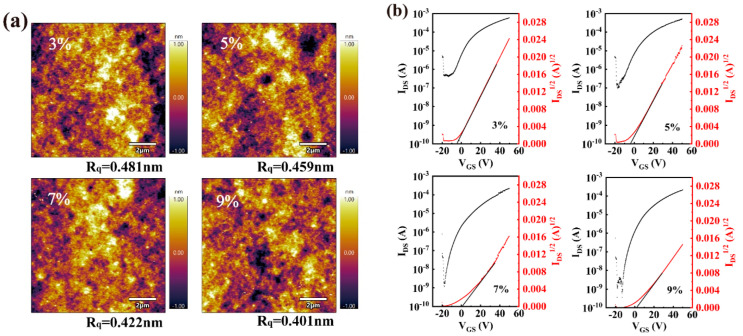
(**a**) AFM images of surface morphology of the In_2_O_3_/InGdO TFTs with different Gd concentrations. (**b**) Typical transfer curves of the In_2_O_3_/InGdO TFTs with different Gd concentrations at V_DS_ = 10 V.

To further optimize the performance of the heterojuction TFTs, we fabricated and investigated In_2_O_3_/InGdO TFTs with different Gd doping concentrations. The morphology of the In_2_O_3_/InGdO thin films was analyzed for understanding the dependence of the properties of the In_2_O_3_/InGdO TFTs on the Gd doping concentration, as shown in Figure 3a. The RMS roughness of the 3%, 5%, 7%, and 9% Gd-doped In_2_O_3_/InGdO films were 0.481 nm, 0.459 nm, 0.422 nm and 0.401 nm, respectively. There was no obvious difference in RMS roughness levels of less than 1 nm, illustrating that the samples are smooth and continuous, which is conducive to strengthening their adhesion and forming a good Ohmic contact between the channel layers and Al source/drain electrodes. Next, the In_2_O_3_/InGdO TFTs with Gd concentrations increasing from 3% to 5%, 7%, and 9% were fabricated and measured, as indicated in Figure 3b. The results are as follows, with accumulating Gd doping concentration: the mobility decreases evidently from 6.22 to 5.38, 4.34, and 2.54 cm^2^V^−1^s^−1^, and the V_th_ gradually rises from −4.61 to −3.77, 0.97, and 2.40 V. Because the oxygen vacancy in the channel layer was suppressed by the Gd ions, less oxygen vacancy which remains in the channel layer ultimately induces a lower conductive In_2_O_3_/InGdO channel. The improvement of the I_on_/I_off_ from 1.84 × 10^3^ to 9.01 × 10^3^, 3.18 × 10^5^, and 3.71 × 10^5^ is owing to the low I_off_ derived from the higher Gd doping concentration in the InGdO layer, and the guaranteed I_on_ originated from the In_2_O_3_ layer thus leads to the increase in I_on_/I_off_. Additionally, the SS varies from 8.10 to 7.68, 4.54, and then to 3.18 V/dec, which proves that the SS of devices can be effectively improved by modulating the buffer layer via changing the concentration of Gd doping ions in the heterojunction channel TFTs.

**Figure 4:**
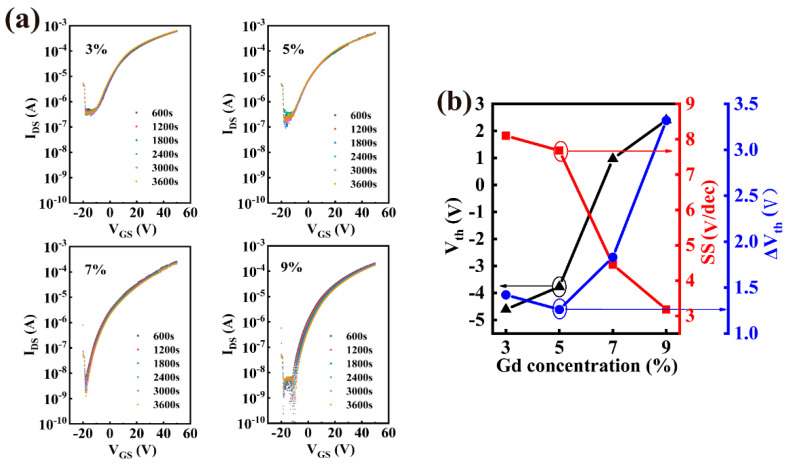
(**a**) The evolution of the transfer cures as a function of applied stress time at V_DS_ = 10 V of the In_2_O_3_/InGdO TFTs with different Gd concentrations. (**b**) The volatility of threshold voltage, subthreshold swing and ΔV_th_ of the In_2_O_3_/InGdO TFTs with increasing Gd concentrations.

The evolution of the transfer curves of the TFTs under the PBS was studied to further investigate the influence of the Gd doping concentration on the stability of In_2_O_3_/InGdO TFTs, as shown in Figure 4a. We found that the ΔV_th_ value decreased slightly when the content of Gd ions increased from 3% to 5%, which is due to fewer oxygen vacancies existing in the channel layer because of the introduction of more Gd ions in higher concentrations of doping samples. The ΔV_th_ value increased again when the content of Gd ions increased from 7% to 9%, and the devices showed a larger ΔV_th_ with stress time. Generally, SS values reflect ΔV_th_, which is related to the fast bulk traps and the interface trap density between the dielectric and the semiconductor [2,23,37]. Thus, we can speculate that additional trap states are generated at the dielectric/semiconductor interface during PBS of TFTs with highly doped semiconductor films or the fast bulk trap density of the device plays a more dominant role than the interfacial trap density [12]. The variations of V_th_, SS and ΔV_th_ are shown in Figure 4b. Additionally, relevant electrical performance parameters are extracted and listed in Table 1. Overall, the In_2_O_3_/InGdO TFTs demonstrate optimum electrical performance and stability at 7% Gd content, which has been verified by several of the experiments.

**Figure 5:**
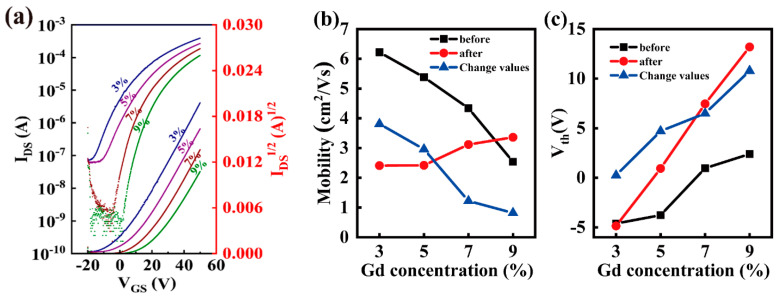
(**a**) Transfer curves of the In_2_O_3_/InGdO TFTs with different Gd doping concentrations at V_DS_ = 10 V after three months. (**b**) The fluctuations of mobility of the In_2_O_3_/InGdO TFTs with different Gd doping concentrations before and after three months. (**c**) The fluctuations of V_th_ of the In_2_O_3_/InGdO TFTs with different Gd doping concentrations before and after three months.

The transfer characteristics as a function of the Gd doping concentration of In_2_O_3_/InGdO TFTs after three months are shown in Figure 5a and associated parameters were extracted and listed in Table 2. It was observed that the devices still have relatively favorable electrical performance compared to the initial one, despite having the larger V_th_. Aging tests have proven that In_2_O_3_/InGdO TFTs can accomplish long-term air stability while maintaining optimal switching property at high mobility. The most intriguing part is that the mobility values of the TFTs increase from 2.41 to 2.42, 3.12, and 3.36 cm^2^V^−1^s^−1^, which is contrary to the increasing doping concentrations from 3% to 5%, 7%, and 9%. Additionally, the V_th_ values for In_2_O_3_/InGdO TFTs changed, with 3%, 5%, 7% and 9% Gd concentrations gradually increased from −4.86 to 0.94, 7.46 and 13.19 V, as shown in Figure 5c. Here, mobility is also associated with a strong increase in V_th_. In addition, we found that the mobility values of the TFTs change from 6.22 to 2.41 cm^2^V^−1^s^−1^ for 3% Gd-doped In_2_O_3_/InGdO TFT, from 5.38 to 2.42 cm^2^V^−1^s^−1^ for 5% Gd-doped In_2_O_3_/InGdO TFT, from 4.34 to 3.12 cm^2^V^−1^s^−1^ for 7% Gd-doped In_2_O_3_/InGdO TFTs, and from 2.54 to 3.36 cm^2^V^−1^s^−1^ for 9% Gd-doped In_2_O_3_/InGdO TFTs after three months. The corresponding values of the mobility change for In_2_O_3_/InGdO TFTs with 3%, 5%, 7% and 9% Gd concentrations gradually diminished from 3.81 to 2.96, 1.22 and 0.82 cm^2^V^−1^s^−1^; the specific trends are shown in Figure 5b. Thereby, we presume that the Gd ions play a key role in inhibiting the aging of the device, although further confirmation is still desirable.

## 4. Conclusions

In conclusion, we fabricated TFTs with the In_2_O_3_/InGdO heterojunction channel layer using a solution process, and compared the electrical properties with single-layer In_2_O_3_ TFTs and InGdO TFTs. In our results, the InGdO TFT showed a higher I_on_/I_off_ of 1.30 × 10^7^, a smaller SS of 1.75 V/dec, and a smaller ΔV_th_ of 0.85 V. Unfortunately, the InGdO TFT achieved a lower mobility of 1.37 cm^2^V^−1^s^−1^ in comparison to the In_2_O_3_ TFT. However, the In_2_O_3_/InGdO TFT, composed of a highly conductive In_2_O_3_ film as the primary transmission layer and a subconductive InGdO film as the buffer layer, showed high electrical performance with higher mobility and higher I_on_/I_off_. The results indicate that the high carrier concentration the In_2_O_3_ layer close to the insulation layer ensures the excellent mobility of In_2_O_3_/InGdO TFTs and low carrier concentration InGdO layer can reduce the I_off_ of the devices, thereby increasing the I_on_/I_off_ of the devices. Furthermore, the PBS test results indicate that TFTs with In_2_O_3_/InGdO heterojunction channels are much more stable. In addition, to further optimize the performance of the heterojunction TFTs, the influence of the Gd doping concentration have been thoroughly studied. The results show the 7% Gd-doped In_2_O_3_/InGdO TFT has optimum characteristics, displaying a superb mobility of 4.34 cm^2^V^−1^s^−1^, a close-to-0 V_th_ of 0.97 V, an SS of 4.54 V/dec, a high I_on_/I_off_ of 3.18 × 10^5^, and a small ΔV_th_ of 1.83 V. Finally, an aging test was carried out after three months, indicating that the In_2_O_3_/InGdO TFTs enable long-term air stability while retaining an optimal switching property of high mobility. The work demonstrates that the heterojunction channel structure of solution-processed metal-oxide semiconductors is feasible for fabricating the high performance TFTs, which further explores a broad pathway for the development of high-performance, low-cost, and large-area oxide electronics.

## Figures and Tables

**Table 1:** Extracted electrical parameters in the In_2_O_3_/InGdO TFTs with various Gd doping concentrations.

Gd Concentration	Saturation Mobility ($cm^{2}$/V s)	Threshold Voltage (V)	Subthreshold Swing (V/dec)	Off-State Current (A)	On/Off Ratio	ΔV_th_ (V)
${\mathrm{In}}_{2}O_{3}$/${\mathrm{In}}_{2}O_{3}$:Gd (3%)	6.22	−4.61	8.10	3.55 × $10^{-7}$	1.84 × $10^{3}$	1.42
${\mathrm{In}}_{2}O_{3}$/${\mathrm{In}}_{2}O_{3}$:Gd (5%)	5.38	−3.77	7.68	7.86 × $10^{-8}$	9.01 × $10^{3}$	1.26
${\mathrm{In}}_{2}O_{3}$/${\mathrm{In}}_{2}O_{3}$:Gd (7%)	4.34	0.97	4.54	1.24 × $10^{-9}$	3.18 × $10^{5}$	1.83
${\mathrm{In}}_{2}O_{3}$/${\mathrm{In}}_{2}O_{3}$:Gd (9%)	2.54	2.40	3.18	9.12 × $10^{-10}$	3.71 × $10^{5}$	3.32

**Table 2:** Extracted electrical parameters in the In_2_O_3_/InGdO TFTs with various Gd doping concentrations after three months.

Gd Concentration	Saturation Mobility ($cm^{2}$/V s)	Threshold Voltage (V)	Subthreshold Swing (V/dec)	Off-State Current (A)	On/Off Ratio
${\mathrm{In}}_{2}O_{3}$/${\mathrm{In}}_{2}O_{3}$:Gd (3%)	2.41	−4.86	8.82	7.54 × $10^{-8}$	8.03 × $10^{3}$
${\mathrm{In}}_{2}O_{3}$/${\mathrm{In}}_{2}O_{3}$:Gd (5%)	2.42	0.94	8.28	5.76 × $10^{-8}$	8.65 × $10^{3}$
${\mathrm{In}}_{2}O_{3}$/${\mathrm{In}}_{2}O_{3}$:Gd (7%)	3.12	7.46	3.93	1.06 × $10^{-9}$	3.44 × $10^{5}$
${\mathrm{In}}_{2}O_{3}$/${\mathrm{In}}_{2}O_{3}$:Gd (9%)	3.36	13.19	3.40	2.96 × $10^{-10}$	8.38 × $10^{5}$

## Data Availability

All data generated or analyzed during this study are included in this published article.
